# Selective effects of conspecific movement on social preference in zebrafish (*Danio rerio*) using real-time 3D tracking and 3D animation

**DOI:** 10.1038/s41598-023-37579-y

**Published:** 2023-06-28

**Authors:** Toshikazu Kuroda, Carolyn M. Ritchey, Christopher A. Podlesnik

**Affiliations:** 1 Huckle Co., Ltd., 2-51 Shiroki, Chikusa, Nagoya, Aichi 464-0846 Japan; 2 Aichi Bunkyo University, 5969-3 Okusa, Komaki, Aichi 485-8565 Japan; 3 Auburn University, 226 Thach Hall, Auburn, AL 36849-5214 USA; 4 Department of Psychology, University of Florida, 945 Center Dr., P.O. Box 112250, Gainesville, FL 32611 USA; 5 Department of Dynamic Brain Imaging, Advanced Telecommunications Research Institute International, 2-2-2 Hikaridai Seika-cho, Kyoto, 619-0288 Japan

**Keywords:** Psychology, Zoology

## Abstract

Zebrafish show social behavior such as shoaling and schooling, which is a result of complex and interdependent interactions among conspecifics. Zebrafish social behavior is interdependent in the sense that one fish’s behavior affects both conspecific behavior and, as a result, their own behavior. Previous research examined effects of the interdependent interactions on the preference for social stimulus but lacked clear evidence that specific conspecific movements were reinforcing. The present research examined whether dependency between individual experimental fish’s motion and a social-stimulus fish’s motions contributes to preference for the social stimulus. In Experiment 1, a 3D animated stimulus fish either chased individual experimental fish or was motionless, serving as dependent and independent motions, respectively. In Experiment 2, the stimulus fish either chased experimental fish, moved away, or moved independently of the experimental fish. In both experiments, experimental fish spent more time near the stimulus fish showing dependent and interactive movements, indicating preference for dependent motion over independent motion, and chasing over other motions. Implications of these results are discussed including a potential role of operant conditioning in the preference for social stimuli.

## Introduction

Zebrafish serve as an animal model in both biomedical and behavioral sciences. The fish have a number of features suitable for biomedical research such as high fecundity, external fertilization, rapid development, and transparency of embryos—an ideal combination for identifying genotype–phenotype relations^1^. Being vertebrates, their nervous system structurally resembles humans compared to invertebrates (e.g., fruit flies), which are traditionally used in genetics research. This structural similarity in turn makes their neurological and pharmacological processes more directly relevant to humans^2–4^. Zebrafish also have a capacity for learning, sharing common behavioral processes with other laboratory species, including rodents and human and nonhuman primates. They acquire involuntary conditioned responses through classical conditioning when stimuli are predictive of food, electric shock, and drugs (e.g.^1,5–7^). Zebrafish also acquire voluntary behavior through operant conditioning when these same stimuli serve as the consequence of behavior (e.g.^8–11^). Thus, zebrafish have high potential for serving as an interface between biomedical and behavioral sciences, including the study of social behavior.

Zebrafish are highly social—when placed together with conspecifics, they engage in shoaling and schooling, often forming hierarchies within a group^12–15^. Shoaling involves fish remaining physically close to one other. Schooling involves swimming together in the same direction, which tends to involve faster motion and less dense cohesion than shoaling and is more likely to occur shortly after being placed in a novel environment than later. Biological factors have been shown to reduce zebrafish social behavior, including genetically modified zebrafish models of autism spectrum disorders (ASD)^16,17^ and embryonic exposure to low doses of ethanol^18^. To better understand how such neurobiological changes affect social behavior, it is necessary to identify how zebrafish generally interact under social conditions.

Characteristics of conspecifics (i.e., stimulus fish) affect the behavior of experimental zebrafish. When placing a transparent partition between the experimental and stimulus fish, male zebrafish showed greater preference to females over males but females approached both sexes similarly^19,20^. For this and subsequent studies, preference was defined as relative time spent in proximity between two social stimuli. The physical appearance of conspecifics also affected preference through computerized manipulations of body color, stripe pattern, and shape of stimulus fish images^21^. Preference was greater for fish images with unaltered color over red, yellow over unaltered images, unaltered images over pixel-scrambled images (stretched, pixelated)^22^, and no preference among different stripe patterns (unaltered/horizontal vs. vertical vs. stripeless).

Motion is another, and more dynamic, characteristic of conspecifics affecting social behavior. One study presented a robotic stimulus fish (i.e., a model attached on an acrylic rod) and based its three-dimensional (3D) motion on a pre-recorded trajectory of live zebrafish^23^. Experimental fish preferred the robotic fish when moving compared to when it remained static. Moreover, another study found that zebrafish preferred a video of an arbitrary stimulus (i.e., dot) mimicking the natural motion of zebrafish over the dot that moved unnaturally, although the fish preferred even more a video of zebrafish naturally swimming^24^. Unlike natural interactions among zebrafish, however, the motions of stimulus fish were independent from movement of experimental fish in both cases. Given the social nature of zebrafish, interactions between fish likely are more dynamic and depend on specific motions when the fish interact.

One relation among fish under natural social conditions is the motion of one fish affects behavior of another fish. In other words, contingencies exist between behavior of conspecifics. One potential contingency is the behavior of the stimulus fish functioning as a consequence of the experimental fish’s behavior through the process of operant conditioning (see^11,17^). For example, some researchers manipulated the correlation between movement of experimental fish and the frequency of a robotic fish’s tail-beating^25^, as tail-beating has been shown to attract experimental fish (see^26^). By tracking the motion of experimental fish in real time, they could manipulate the frequency of tail-beating based on the motion of experimental fish. In a within-subjects comparison, experimental fish spent more time near the stimulus fish when doing so led to faster tail-beating (positive correlation) than when it led to slower tail-beating (negative correlation). Nonetheless, time spent near the stimulus fish in the positive-correlation condition did not differ from uncorrelated conditions in which the tail-beating frequency either remained constant or varied independently from the experimental fish’s motion. Thus, it remains unclear the extent to which the dependency between zebrafish behavior and changes in conspecific behavior directly affect behavior through operant contingencies.

The goal of the present research was to examine whether and how the dependency between experimental- and stimulus-fish movement affects preference as relative time spent near either of two stimulus fish, with each stimulus fish displaying a different type of motion on separate monitors. Across two experiments, we examined the preference between stimulus fish engaging in motion-dependent chasing of experimental fish versus (1) being motionless or (2) engaging in different types of behavior-dependent chasing versus fleeing, as well as a comparison of behavior-dependent and -independent motions. We displayed animated 3D stimulus fish developed from a real zebrafish photo, thereby appearing more natural and without unnatural robotic noises when compared with robotic fish used in previous research (e.g.^23,25^). The motion and orientation could in 3D allowed stimulus fish to be precisely and automatically controlled in real time in reference to the motion of an experimental fish measured using a tracking system^27^. We expected greater preference for stimulus fish engaging in movements dependent on experimental-fish behavior if operant behavior underlies engagement in at least some aspect of zebrafish social behavior.

## Experiment 1

We examined preference between motion-dependent, motionless, and the absence of a stimulus fish. Experiment 1 was a systematic replication of prior research^23^ in which zebrafish preferred a moving robotic stimulus fish over its motionless counterpart. We extended their study in two respects. First, we used a 3D-fish animation as the stimulus fish compared with their use of a robotic fish. Second, the present experiment arranged that motion of the stimulus fish was dependent on specific movements of the experimental fish but was dependent only on the proximity between the two fish in Ref.^23^. The goal of the present experiment was to validate our novel methods by assessing whether our findings replicated the preference for moving over motionless stimulus fish observed in Ref.^23^.

### Methods

#### Subjects

Four male and four female experimentally naïve zebrafish (National Bioresource Project of Japan, Riken Brain Science Institute), 8–10 months old at the start of the experiments, served as subjects. They were wild type and not genetically modified. From two weeks before the experiment to its completion, each was housed individually in a room with a 14 h:10 h light–dark cycle (lights on at 7:00 am) in a home aquarium (13-cm wide by 17-cm long by 11-cm high) connected to a pump and tank where water was filtered, aerated, and maintained at 28 °C with a heater. The fish could see each other in the home aquariums. Water was kept at a pH level of 7.5 and half of the water in the tank was replaced weekly. Post-session feedings (Kyorin, Hikari Labo 270) occurred 30 min after sessions. This research was approved by the Research Ethics Committee at Aichi Bunkyo University, all the experiments reported herein were performed in accordance with animal research regulations in Japan, and the present authors complied with the ARRIVE guidelines.

#### Apparatus

**Figure 1:**
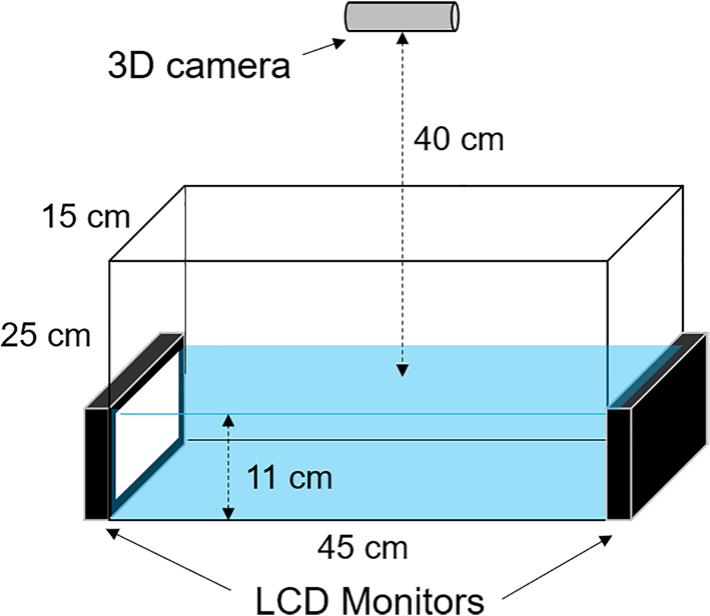
A diagram of the apparatus. See the main text for details.

Experimental aquariums (15-cm wide × 45-cm long × 25-cm high) had a glass wall on each side and a white plastic board at the bottom (see Fig. 1). Water was filled to 11 cm high. The water temperature was set at 28 °C at the onset of each session. Two LCD monitors (Elecrow®) having a 15-cm wide by 8.5-cm high screen covered left and right walls of the aquarium. The top of the screen matched the level of the water surface. A 3D camera (Intel® RealSense™ D435 model) was placed 40 cm above, and parallel to, the water surface. The camera generated a set of color and depth frames which, in turn, went through a series of image processing for tracking the motion of the fish in real time (see “Motion tracking” section for details). A white LED light (265 lumens) was placed above the camera providing general illumination. The aquarium was place on a metal rack and the whole rack was covered with a gray curtain to minimize visual distraction during sessions.

A C++ program operating on a custom-built desktop computer controlled all devices and recorded all experimental events. The computer, running Ubuntu 18.04 operating system, was built with a motherboard (ASUS® ROG Maximus Xi Code), a CPU (Intel® Core i7 9700K), a GPU (NVIDIA® Titan RTX), two RAMs (G.Skill® DDR4-4000), and a hard drive (Samsung® 970EVO Plus SSD).

#### Social stimuli

3D computer-animated zebrafish served as the social stimuli. A photo of real female zebrafish, available from https://commons.wikimedia.org/wiki/File:Zebrafisch.jpg, was converted to a 3D image (.obj file) using an online tool (https://monstermash.zone/) (see^28^ for details). We used the female photo based on a previous finding that both male and female zebrafish approach female conspecifics^19,20^. The rotation and location of the stimulus fish on a monitor were controlled in real time using an OpenGL-based library (https://github.com/WHKnightZ/OpenGL-Load-Model). The body size of the stimulus fish was set constant at 3 cm long from a side view, approximately the same size as experimental fish.

**Figure 2:**
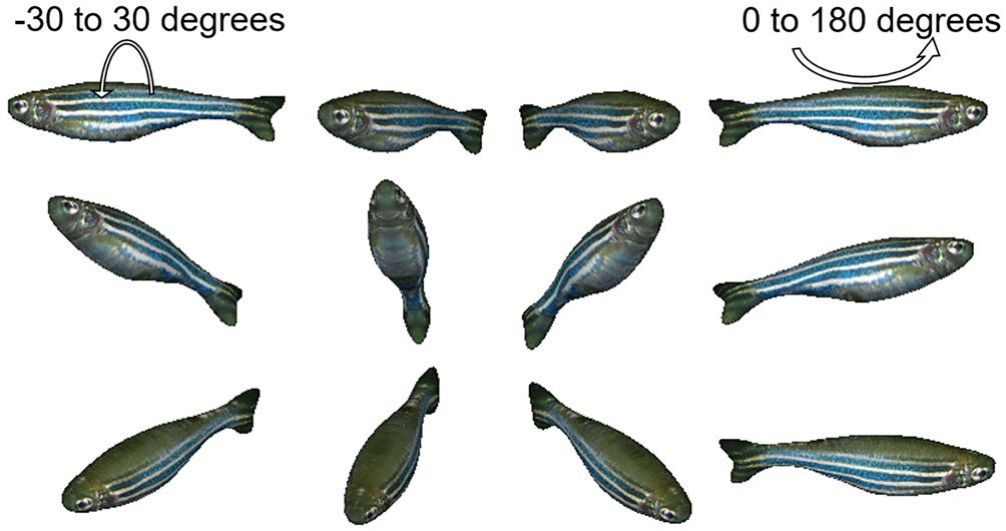
Sample images of the 3D animation for various orientations. Fins were removed during the conversion process from the original 2D image to the 3D image because they inflated and looked unreal. The horizontal rotation was limited between 0 and 180 degrees while the vertical rotation was limited between − 30 and 30 degrees. The 3D image was made from a photo of real female zebrafish (https://commons.wikimedia.org/wiki/File:Zebrafisch.jpg) using an online tool (https://monstermash.zone/).

Restrictions were set on the stimulus fish rotation (see Fig. 2). Thus, its horizontal rotation was limited between 0 and 180 degrees (the head orienting leftward and rightward, respectively), keeping the head orienting toward the experimental fish. Its vertical rotation was limited between − 30 and 30 degrees, keeping its back upward and its abdomen downward. With these restrictions on its motion, the stimulus fish showed different types of motion depending on experimental conditions as described in detail below.

#### Motion tracking

A set of color and depth frames (640-pixel width × 360-pixel height each) streamed from the camera was processed for measuring 3D coordinates of a fish (*x, y,* and* z* values in meters relative to the camera)^27^. Two different deep-learning algorithms were simultaneously used for detecting experimental fish and their body parts on color frames. One was YOLO (“You only look once”)^29^, a widely used real-time object-detection algorithm for its well-balanced processing speed and detection accuracy. The other was DeepLabCut (ver. 2.1)^30^, an algorithm for detecting body parts. Hundreds of photos with manually annotated zebrafish were used for training each deep-learning model. The rationale for using the two different algorithms was to minimize false detections during real-time tracking. YOLO created a bounding box around a fish. Then the detection of its body parts by DeepLabCut was considered valid only if those parts were inside the YOLO bounding box. This requirement led to almost complete absence of false detections with only occasional false negatives. Overall, approximately 30 sets of color and depth frames were processed per s (30 FPS), being equivalent to the processing speed of conventional webcams. For analyzing motion of experimental fish, the head was considered the fish’s location at all times although the body and the tail also were tracked and recorded.

### Procedure

Sessions were conducted individually for each fish daily, around the same time of the day, seven days a week. One day before the first session, the fish were placed in the aquarium for a 20-min acclimatization period. Thereafter, each session lasted for 20 min and was divided into four 5-min periods (a presession period and Periods 1–3). The entire screen of both LCD monitors remained white in the presession period and Periods 1, and 3. The presession period was for minimizing effects of transporting the fish with a Kyorin brand “water net” that holds water and allows for gentle transportation from the home aquarium to the experimental aquarium. Periods 1 and 3 served as baselines during which no stimulus fish were presented. Thus, experimental conditions, as described in detail below, differed in how we arranged stimulus fish during Period 2.

**Figure 3:**
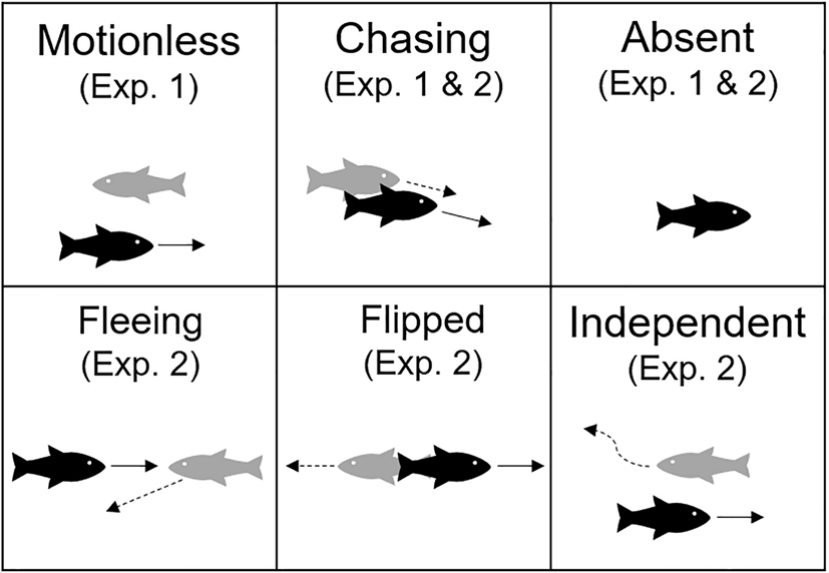
Diagrams for each motion type used in the present research. The fish in black and in gray represent experimental and stimulus fish, respectively. The motion of the experimental fish (solid arrow) is followed by the motion of the stimulus fish (dotted arrow). See the main text for details.

The top row of Fig. 3 shows a diagram for each type of stimulus presented on a monitor during Period 2 in this experiment with a sample video for each type being available from https://github.com/ToshiEAB/social_stimulus_preference). Three types of stimuli were examined in this experiment.

#### Motionless

The first type of stimulus was *motionless*. It was motionless in the sense that stimulus fish stayed at the center of monitor with its head orientating leftward. Nonetheless, it was not completely still: It moved leftward or rightward by 0 to 2 pixels (randomly selected) every 0.033 s (i.e., 30 times per s) from the center of the monitor to simulate the idling motion of live fish.

#### Chasing

The second type of stimulus was *chasing*. Here, the stimulus fish followed the experimental fish whenever the latter moved: When the experimental fish’s head moved one direction, the stimulus fish also moved the same direction in a similar way to mirror images (but the motion onset was delayed until the distance between the heads of two fish was greater than 16 pixels), regardless of whether the two fish were on the same or opposite side of the aquarium. The motion velocity was positively correlated with the distance between their heads. Specifically, the distance in pixel was calculated using the Pythagorean theorem assuming that the two fish were on the same 2D plane. The motion velocity at a given moment was calculated by dividing the distance in pixel by a constant value of 650. The velocity was updated every 0.033 s, resulting in velocities similar to those of live zebrafish motion. Overall, the velocity generally was the highest when stimulus fish started moving and then decreased as it approached the experimental fish without sudden stops.

#### Absent

The third type of stimulus was *absent*, displaying no stimulus fish on a monitor. Although being absent technically was not a type of motion, it will be described as such in the present research for the sake of simplicity. For all motion types, the background color always was white.

Table 1 shows a summary of the order of experimental conditions for each fish. Briefly, the three types of stimuli described in the preceding paragraph were compared to each other. All conditions consisted of eight consecutive sessions with stimulus displays reversing after the fourth session. For four experimental fish (two males and two females), the first four sessions consisted of one stimulus (e.g., chasing) on the left monitor and a different stimulus (e.g., motionless) on the right monitor, with the sides being reversed during the remaining four sessions. The sides presenting the stimuli across sessions were opposite for the remaining four fish. An exception to these rules was made to the Absent-vs-Absent condition, which served as a control for all other conditions. During these conditions, two consecutive sessions in which no stimuli appeared on either monitor throughout were conducted before the first experimental condition and after each experimental condition, for a total of eight sessions. These interspersed control sessions together constituted the Absent-vs-Absent condition. (Note: Another Absent-vs-Absent condition consisting of eight consecutive sessions was conducted after Experiment 2, resulting in virtually no difference from the interspersed sessions. Thus, the latter data were used for analyses here.)

**Table 1:** The order of experimental conditions for each fish in Experiment 1.

Conditions	Fish ID			
	1M and 3F	2M and 4F	5M and 7F	6M and 8F
Motionless vs. Absent				
Motionless on the left	1	2	3	4
Motionless on the right	2	1	4	3
Chasing vs. Absent				
Chasing on the left	3	4	1	2
Chasing on the right	4	3	2	1
Chasing vs. Motionless				
Chasing on the left	5	6	5	6
Chasing on the right	6	5	6	5
Absent vs. Absent	(Interspersed across conditions)			

See the main text for details of how the Absent-vs-Absent condition was conducted.

### Data analysis

The 45-cm long aquarium was equally divided into left, center, and right compartments (15-cm long each) and time spent in the left and right compartments was the primary measure for data analyses (e.g.^21^). We did not include time spent in the center compartment in our formal analyses because its implications for preference are ambiguous, as it could indicate either transitions between left and right compartments or no preference. Time in the center compartment is available as supplementary material. Separately for Periods 1–3, the time spent in the compartment associated with one stimulus (e.g., motion) was summed across eight sessions in a condition, likewise for the other stimulus (e.g., motionless), and the ratio of time was calculated as the index of preference. This index controlled for position bias because each motion type was associated with the left compartment for four sessions and with the right compartment for the remaining four sessions. In the Absent-vs-Absent condition, during which eight sessions (i.e., four sets of two consecutive sessions) were interspersed across conditions, we controlled for position bias by having one stimulus treated as if being associated with the left compartment in the first one of the two sessions in each set and with the right compartment in the second session.

A logarithmic transformation was applied to the time ratio (preference) to (1) allow for direct comparisons of preference quantitatively in either direction and (2) normalize distributions of means around 0 for statistical analyses. After obtaining log ratios individually for each fish, arithmetic means were calculated within each condition. Following statistical analysis, the log ratio of preference was transformed back to its original ratio for interpreting the results. As a consequence, arithmetic mean log ratio turned into geometric mean of the original ratio, which is a more appropriate measure of central tendency when original measures always are positive values and skewed to the right^31^, as in the present case.

We used a mixed-effects modeling approach to evaluate effects of experimental contingencies on log ratios of preference. This approach preserves individual-level variability and accounts for intercorrelations at the subject and group level. We completed this analysis using the R Statistical Program and the lme method contained in the *lme4* package^32^. The analysis comprised several steps. We first evaluated the random-effects structure using the second-order Akaike Information Criterion (AICc) with up to two simultaneous random-slope effects—i.e., Period (1–3) and Condition (Motionless-vs-Absent, Chasing-vs-Absent, Chasing-vs-Motionless, and Absent-vs-Absent). These random-slope effects allowed differences for individual fish with respect to changes in preference periods and experimental conditions. We performed model comparisons using AICc via the *MuMIn* R package before evaluating fixed effects using likelihood-ratio tests. Finally, using the *emmeans* package^33^, we conducted post hoc comparisons of preference (1) between conditions within each period and (2) between periods within each condition. We did not include sex as a between-subjects factor because of the small sample size (four males and four females). To account for multiple comparisons, we report adjusted *p* values calculated using the Holm-Bonferroni method^34^.

### Results and discussion

**Figure 4:**
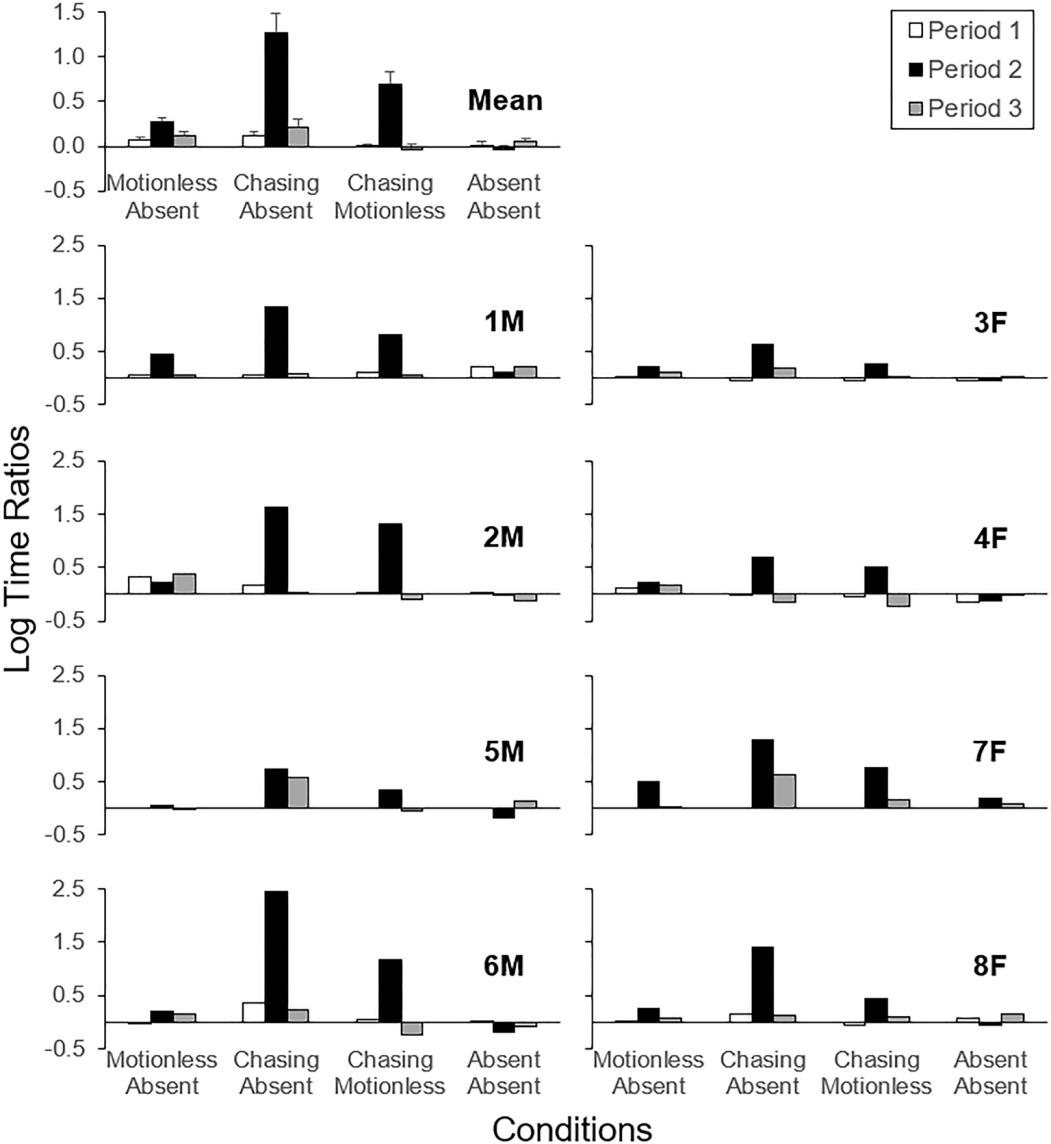
Log ratios of time spent in the side compartments of the aquarium across Periods 1–3 (white, black, and gray bars; 5 min each) for each subject in each condition in Experiment 1, with the arithmetic mean log ratios on the top left corner. Positive and negative values respectively indicate more time spent in the compartment associated with the first and second motion type in the name of condition. For instance, motionless and absent correspond to the first and second motion type in the Motionless-vs-Absent condition. Labels (e.g., “1 M”) indicate subject IDs where M and F represents male and female, respectively. Error bars represent standard errors.

Figure 4 shows log time ratios in Periods 1–3 in each condition individually for each fish, with the arithmetic mean log ratio averaged across fish on the top left corner. In the Motionless-vs-Absent condition, log ratios were slightly to moderately higher in Period 2 than other Periods for all but one fish (2M). In the Chasing-vs-Absent condition, log ratios were consistently the highest in Period 2 for all fish. In the Chasing-vs-Motionless condition, all fish showed highest log ratios in Period 2, indicating preference for the chasing motion over motionless. Log ratios in the Absent-vs-Absent condition were close to zero in most cases and unsystematic across Periods 1–3, suggesting that time passage per se within a session had no systematic effect on the preference measure.

Table 2 shows the results of the statistical analysis, with Period (2) and Condition (Absent-vs-Absent) factors serving as individual contrasts. There were significant interactions between Period and Condition (*p*s < 0.0001) indicating a different effect of Period (1 and 3 vs. 2) in the Chasing-vs-Absent and Chasing-vs-Motionless conditions compared to the Absent-vs-Absent condition. Post hoc comparisons within Period 2 indicated higher log time ratios (1) in each condition compared to Absent-vs-Absent (*p*s ≤ 0.004), (2) in Chasing-vs-Absent compared to Chasing-vs-Motionless and Motionless-vs-Absent (*p*s < 0.0001) and (3) in Chasing-vs-Motionless compared to Motionless-vs-Absent. Table 3 shows estimated marginal means for each of these conditions within Period 2. None of the between-condition comparisons within Period 1 and 3 reached statistical significance (*p*s ≥ 0.113). Post hoc comparisons also indicated higher log time ratios in Period 2 compared to 1 and 3 within the Chasing-vs-Absent and Chasing-vs-Motionless conditions (*p*s ≤ 0.0001)—see Table 3 for details. Those comparisons did not reach statistical significance within the Motionless-vs-Absent and Absent-vs-Absent conditions (*p*s ≥ 0.301). Comparisons between Periods 1 and 3 also did not reach statistical significance within any condition (*p*s ≥ 0.339). Overall, these statistical results were consistent with the above interpretations where data were analyzed individually for each fish: Zebrafish preferred the chasing motion to a motionless stimulus fish. Numerically, the arithmetic mean log time ratio was 0.70 in Period 2 of the Chasing-vs-Motionless condition. Its antilog (5.02) indicated that, on average, zebrafish preferred the chasing motion approximately five times more than motionless fish.

**Table 2:** Results of linear mixed-effects regression, Experiment 1.

Factor	β (SE)	df	*t*	*p*
Intercept	− 0.04 (0.11)	15.02	− 0.33	0.748
Period (1)	0.05 (0.12)	31.68	0.44	0.664
Period (3)	0.09 (0.13)	21.61	0.66	0.517
Condition (Chasing-vs-Absent)	1.31 (0.10)	77.00	12.86	< 0.001
Condition (Chasing-vs-Motionless)	0.74 (0.10)	77.00	7.25	< 0.001
Condition (Motionless-vs-Absent)	0.30 (0.10)	77.00	2.98	0.004
Period (1) × Condition (Chasing-vs-Absent)	− 1.21 (0.14)	77.00	− 8.43	< 0.001
Period (3) × Condition (Chasing-vs-Absent)	− 1.14 (0.14)	77.00	− 7.97	< 0.001
Period (1) × Condition (Chasing-vs-Motionless)	− 0.75 (0.14)	77.00	− 5.24	< 0.001
Period (3) × Condition (Chasing-vs-Motionless)	− 0.82 (0.14)	77.00	− 5.71	< 0.001
Period (1) × Condition (Motionless-vs-Absent)	− 0.25 (0.14)	77.00	− 1.78	0.080
Period (3) × Condition (Motionless-vs-Absent)	− 0.23 (0.14)	77.00	− 1.62	0.109

The Period (2) and Condition (Absent-vs-Absent) factors served as individual contrasts.

**Table 3:** Estimated marginal means, Experiment 1.

Period/condition	emmean (SE)	df	95% CI
Condition within Period 2			
Absent-vs-Absent	− 0.04 (0.11)	14.90	− 0.27, 0.20
Chasing-vs-Absent	1.27 (0.11)	14.90	1.04, 1.50
Chasing-vs-Motionless	0.70 (0.11)	14.90	0.47, 0.93
Motionless-vs-Absent	0.27 (0.11)	14.90	0.03, 0.50
Period within chasing-vs-Absent condition			
1	0.11 (0.07)	48.10	− 0.04, 0.26
2	1.27 (0.11)	14.90	1.04, 1.50
3	0.21 (0.07)	55.90	0.07, 0.36
Period within chasing-vs-Motionless condition			
1	0.00 (0.07)	48.10	− 0.15, 0.15
2	0.70 (0.11)	14.90	0.47, 0.93
3	− 0.03 (0.07)	55.90	− 0.18, 0.11

*emmean* estimated marginal mean.

The present results were consistent with the finding in a previous study that zebrafish preferred stimulus fish engaging in motion over motionless fish despite differences in the experimental stimuli and procedure^23^. As for the stimuli, these researchers used a robot as a stimulus fish whereas we used a 3D animation. The use of animated 3D fish could be useful for future research on zebrafish social behavior, as it eliminates any unnatural sounds resulting from the mechanics of robotic fish and offers a wider variety of possible movements. Although we have not provided definitive evidence as to which of the methods is better suited for the analysis of zebrafish social behavior, consistent results across the two studies indicate the robustness of the results regarding the effects of motion on preference. As for the procedure, the researchers only assessed independent effects of each motion type, analogous to the present Motionless-vs-Absent and Chasing-vs-Absent conditions^23^. In contrast, we also included a direct comparison of those effects in the Chasing-vs-Motionless condition. The latter allowed for a quantification of the relative preference between two different stimulus fish (e.g., the chasing motion was preferred five times greater than motionless) while controlling all other variables. To our knowledge, this was the first study in which a hierarchy of preference among social stimuli in zebrafish were defined quantitatively.

## Experiment 2

Experiment 1 demonstrated a strong preference for stimulus fish engaging in a chasing motion dependent on the experimental fish’s motion compared with a motionless stimulus fish. In the present experiment, we addressed two main goals by examining different aspects of the dependency between the motions of experimental and stimulus fish. The first goal was to examine how variations in type of dependent motion (e.g., chasing vs. fleeing) affect preference for social stimuli. A second goal was to examine effects of the motion-dependency on preference by maintaining versus removing the dependency. As for the latter goal, a previous study did not find clear effects of the motion-dependency when correlating the frequency of tail-beating of a robotic fish to the distance between experimental and robotic fish^25^. However, the robotic tail beating might have introduced other factors making it challenging to isolate the role of dependency. For example, the visual and auditory effects of tail-beating from the robotic fish could be aversive and potentially to greater extents at higher frequencies. Therefore, the present experiment used a 3D animated stimulus fish to examine whether motion-dependency contributes to preference for social stimuli.

### Subjects and apparatus

The eight zebrafish served in the first experiment also served in this experiment. The apparatus was as described in the first experiment.

### Procedure

General features of the experimental procedure were as in the first experiment. A 20-min session was divided into four 5-min periods (a presession period and Periods 1–3) and a stimulus fish was presented on each monitor only in Period 2.

Figure 3 shows a diagram for each of the five stimulus types used in this experiment.

#### Chasing

Same as described in Experiment 1.

#### Absent

Same as described in Experiment 1.

#### Fleeing

*Fleeing* was dependent on the movement of experimental fish but differed from chasing in that stimulus fish moved away from the experimental fish. Specifically, stimulus fish moved to a different part of the monitor when experimental fish approached it, where approach was defined as that the distance between their heads was within 350 pixels horizontally and 150 pixels vertically (approximately 5.2 cm and 2.1 cm, respectively). If the stimulus fish was on the left side of the monitor when the approach occurred, the fish moved to a random location on the right side; if the stimulus fish was on the right side, it moved to a random location on the left side.

#### Flipped

*Flipped* was a mirror image of the chasing motion. This stimulus was included as a control to compare with all aspects of chasing, including motion onset and speed. The only difference was the reversed left/right directions while maintaining the other features of fleeing.

#### Independent

*Independent* arranged for the stimulus fish to move independently from the movement of experimental fish. Specifically, a 5-min long video was played on a monitor. The video was one of four recordings of a stimulus fish chasing a zebrafish that did not serve in these experiments. All videos were presented in the same order for all fish.

Table 4 shows a summary of the order of experimental conditions for each fish. In general, the chasing motion was compared with fleeing, flipped, and independent motions. Each condition included eight consecutive sessions. In the first four sessions for half of the fish (two males and two females), one stimulus was presented on the left monitor while a different stimulus was presented on the right monitor, with sides being reversed during the next four sessions. The other half of the experimental fish received reversed exposure to the different stimuli across the two monitors. An exception was made to the Absent-vs-Absent condition. As in Experiment 1, two consecutive sessions in which no social stimulation occurred were interspersed across other experimental conditions, for a total of eight sessions.

**Table 4:** The order of experimental conditions for each fish in Experiment 2.

Conditions	Fish ID	
	1M, 3F, 5M, and 7F	2M, 4F, 6M, 8F
Chasing vs. Fleeing (1)		
Chasing on the left	1	2
Chasing on the right	2	1
Chasing vs. Flipped		
Chasing on the left	3	4
Chasing on the right	4	3
Chasing vs. Independent		
Chasing on the left	5	6
Chasing on the right	6	5
Chasing vs. Fleeing (2)		
Chasing on the left	7	8
Chasing on the right	8	7
Absent vs. Absent	(Interspersed across conditions)	

See the main text for details of how the Absent-vs-Absent condition was conducted.

### Data analysis

The general strategy for data analysis was as described in the first experiment, except that we evaluated the random-effects structure using the second-order AICc with up to two simultaneous random-slope effects: Period (1–3) and Condition (Chasing-vs-Flipped, Chasing-vs-Independent, Chasing-vs-Fleeing-1, and Chasing-vs-Fleeing-2).

### Results and discussion

**Figure 5:**
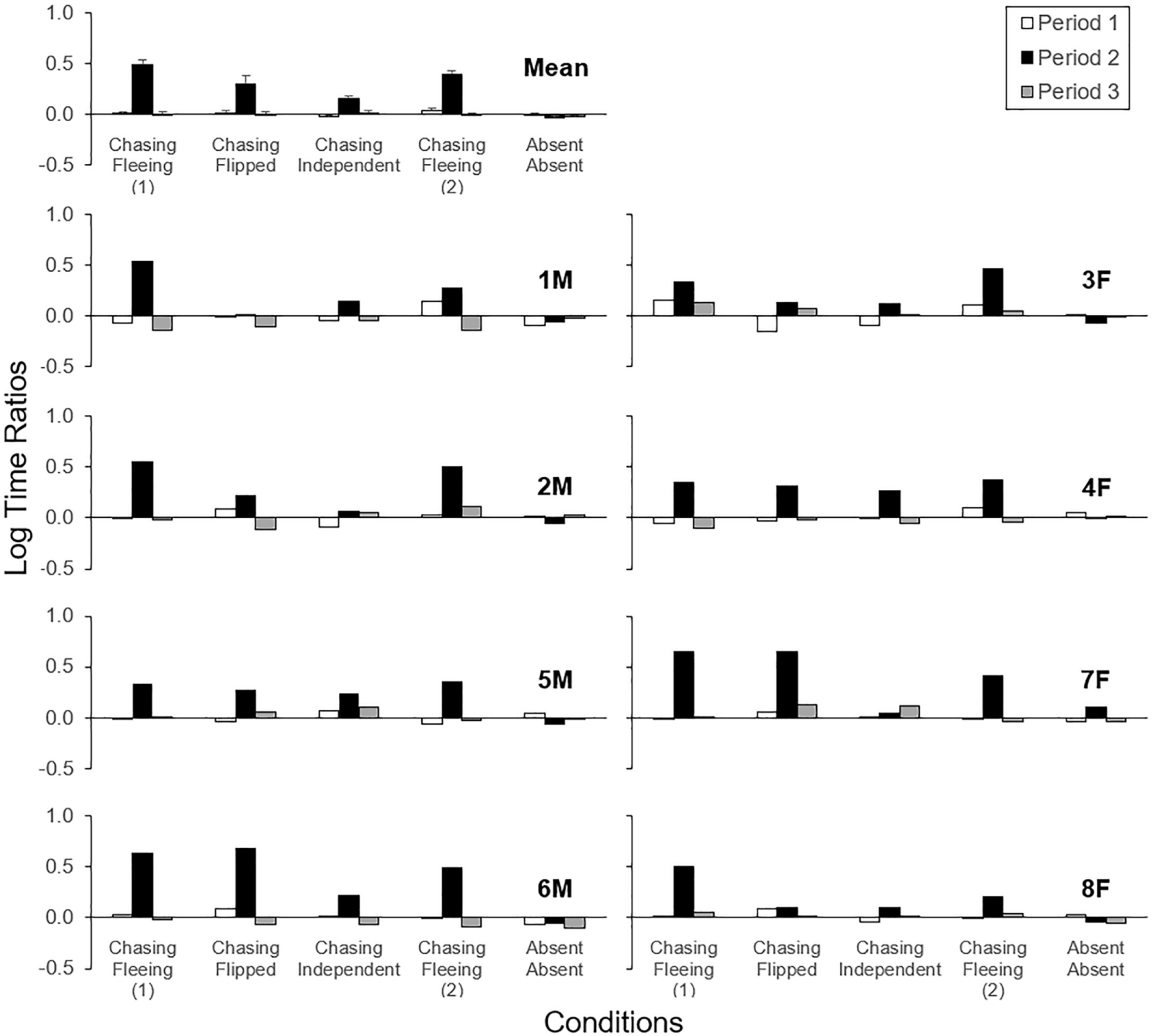
Log ratios of time spent in the side compartments of the aquarium across Periods 1–3 (white, black, and gray bars; 5 min each) for each subject in each condition in Experiment 2, with the arithmetic mean log ratios on the top left corner. Other details are as described in Fig. 4.

Figure 5 shows log time ratios in Periods 1–3 in each condition individually for each fish, with the arithmetic mean log ratio on the top left corner. In the Chasing-vs-Fleeing condition for both the first and second assessments, all subjects showed highest log ratios in Period 2. In the Chasing-vs-Flipped condition, all fish showed highest log ratios in Period 2, though the differences were relatively small in two fish (1M and 8F). In the Chasing-vs-Independent condition, most fish showed highest log ratios in Period 2 though differences were relatively small for one fish (2M) and another fish (7F) showed the highest log ratio in Period 3. Finally, log ratios were close to zero and were unsystematic across Periods 1–3 in the Absent-vs-Absent condition.

Table 5 shows the results of the statistical analysis, with Period (2) and Condition (Absent-vs-Absent) factors again serving as individual contrasts. There were significant interactions between Period and Condition (*p*s < 0.039), indicating a different effect of Period (1 and 3 vs. 2) in each condition compared to the Absent-vs-Absent condition. Post hoc comparisons within Period 2 indicated higher log time ratios (1) in each condition compared to Absent-vs-Absent (*p*s ≤ 0.001), (2) in Chasing-vs-Flipped compared to Chasing-vs-Independent (*p* = 0.010), (3) in Chasing-vs-Fleeing-1 compared to Chasing-vs-Flipped (*p* = 0.001) and (4) in both Chasing-vs-Fleeing conditions compared to Chasing-vs-Independent (*p*s ≤ 0.001). Table 6 lists estimated marginal means for each condition within Period 2. The remaining comparisons in Period 2 (Chasing-vs-Flipped and Chasing-vs-Fleeing-2, Chasing-vs-Fleeing-1 and -2) did not reach statistical significance (*p*s = 0.098); this was also true of the between-condition comparisons within Period 1 and 3 (*p*s = 1.00). Post hoc comparisons also indicated higher log time ratios in Period 2 compared to 1 and 3 within each condition except for Absent-vs-Absent (*p*s ≤ 0.015; Absent-vs-Absent: *p*s = 1.00)—see Table 6 for details. Comparisons between periods 1 and 3 did not reach statistical significance within any condition (*p*s ≥ 0.298). Overall, these results indicate that zebrafish prefer the chasing motion to other dependent-type motions and to independent motions. The mean differences in log time ratio were 0.49, 0.30, 0.15, and 0.39 for Chasing-vs-Fleeing-1, Chasing-vs-Flipped, Chasing-vs-Independent, and Chasing-vs-Fleeing-2, respectively. These correspond to 3.07, 1.99, 1.41, and 2.45-fold differences in time ratio in that order.

**Table 5:** Results of linear mixed-effects regression, Experiment 2.

Factor	β (SE)	df	*t*	*p*
Intercept	− 0.03 (0.04)	102.65	− 0.91	0.365
Period (1)	0.03 (0.05)	98.00	0.57	0.573
Period (3)	0.01 (0.05)	98.00	0.22	0.825
Condition (Chasing-vs-Flipped)	0.33 (0.05)	98.00	6.71	< 0.001
Condition (Chasing-vs-Independent)	0.18 (0.05)	98.00	3.71	< 0.001
Condition (Chasing-vs-Fleeing-1)	0.52 (0.05)	98.00	10.53	< 0.001
Condition (Chasing-vs-Fleeing-2)	0.42 (0.05)	98.00	8.54	< 0.001
Period (1) × Condition (Chasing-vs-Flipped)	− 0.32 (0.07)	98.00	− 4.52	< 0.001
Period (3) × Condition (Chasing-vs-Flipped)	− 0.31 (0.07)	98.00	− 4.49	< 0.001
Period (1) × Condition (Chasing-vs-Independent)	− 0.20 (0.07)	98.00	− 2.92	0.004
Period (3) × Condition (Chasing-vs-Independent)	− 0.15 (0.07)	98.00	− 2.10	0.039
Period (1) × Condition (Chasing-vs-Fleeing-1)	− 0.51 (0.07)	98.00	− 7.30	< 0.001
Period (3) × Condition (Chasing-vs-Fleeing-1)	− 0.51 (0.07)	98.00	− 7.26	< 0.001
Period (1) × Condition (Chasing-vs-Fleeing-2)	− 0.38 (0.07)	98.00	− 5.45	< 0.001
Period (3) × Condition (Chasing-vs-Fleeing-2)	− 0.41 (0.07)	98.00	− 5.94	< 0.001

The Period (2) and Condition (Absent-vs-Absent) factors served as individual contrasts.

**Table 6:** Estimated marginal means, Experiment 2.

Period/condition	emmean (SE)	df	95% CI
Condition within Period 2			
Absent-vs-Absent	− 0.03 (0.04)	103.00	− 0.10, 0.04
Chasing-vs-Flipped	0.30 (0.04)	103.00	0.23, 0.37
Chasing-vs-Independent	0.15 (0.04)	103.00	0.08, 0.22
Chasing-vs-Fleeing-1	0.49 (0.04)	103.00	0.42, 0.56
Chasing-vs-Fleeing-2	0.39 (0.04)	103.00	0.32, 0.46
Period within chasing-vs-Flipped condition			
1	0.01 (0.04)	103.00	− 0.06, 0.08
2	0.30 (0.04)	103.00	0.23, 0.37
3	− 0.00 (0.04)	103.00	− 0.07, 0.07
Period within chasing-vs-Independent condition			
1	− 0.03 (0.04)	103.00	− 0.10, 0.05
2	0.15 (0.04)	103.00	0.08, 0.22
3	0.02 (0.04)	103.00	− 0.06, 0.09
Period within chasing-vs-Fleeing-1 condition			
1	0.01 (0.04)	103.00	− 0.06, 0.08
2	0.49 (0.04)	103.00	0.42, 0.56
3	− 0.01 (0.04)	103.00	− 0.08, 0.06
Period within chasing-vs-Fleeing-2 condition			
1	0.04 (0.04)	103.00	− 0.03, 0.11
2	0.39 (0.04)	103.00	0.32, 0.46
3	− 0.01 (0.04)	103.00	− 0.09, 0.06

*Emmean* estimated marginal mean.

The findings of the present experiment suggest zebrafish prefer certain response-dependent movements of conspecifics over others. Specifically, chasing was preferred over both the flipped and, to a greater degree, the fleeing motion. The flipped motion differed from the chasing motion only in the direction of stimulus fish’s motion in reference to the experimental fish’s motion, thereby controlling for many potential quantitative differences in stimulus-fish movement (e.g., motion-dependency, motion speed, motion onsets). In contrast, the fleeing motion allowed for more variation in motion patterns (e.g., speed and onset) than the flipped motion. Nevertheless, further research is needed to identify the mechanisms and specific characteristics underlying preference being greatest for the chasing motion.

To our knowledge, this experiment provided the strongest evidence of a reinforcing effect of dependency between the motions of experimental and stimulus fish on the preference for social stimuli in zebrafish. A previous study provided equivocal evidence^25^ but there were numerous differences in methods from the present experiment. First, physical aspects of the stimulus fish differed considerably. The 3D animated fish arranged in the present experiments closely approximated real zebrafish while the robotic fish was approximately five times larger than typical adult zebrafish with numerous aesthetic differences (e.g., shape, stripe patterns), with such features of the robot potentially undermining preference (e.g.^22^). In fact, the robotic fish is not preferred to conspecifics and appears not to be recognized as a conspecific^26^. Thus, preference is comprised of both the conspecific as a stimulus and type of movement (see also^24^). Second, the contingencies differed between experiments, with proximity-dependent tail-beating of the robotic fish versus direction-dependent movement of the 3D animation. Third, approach was assessed to one type of motion of the robotic fish at a time while we assessed preference between two types of movement. Any of these differences in methods could have contributed to the different findings between experiments.

## General discussion

The present research examined how the dependency between experimental- and stimulus-fish motion contributed to preference in zebrafish. Experiment 1 showed that zebrafish preferred a moving conspecific (i.e., chasing) over the absence of motion (i.e., motionless). Experiment 2 revealed a preference for dependency between motion of the experimental and stimulus fish over movements independent of the experimental fish. Finally, we found greater preference for the chasing motion over other forms of dependent motion (fleeing, flipped). Taken together, these experiments indicate a preference for what might be called “interactive” social stimuli. These relations became evident by using a 3D zebrafish animation, which has potential for arranging a wide variety of social stimuli and contingencies.

The present research extended previous findings of stimulus-fish motion on preference in several ways. First, to our knowledge, it provided the first evidence that motion dependency in conspecifics contributes to preference in zebrafish. A previous study examined this possibility by correlating the frequency of a robotic fish’s tail-beating with an experimental fish’s motion^25^. Preference for the stimulus fish was stronger with a positive than a negative correlation but was no different from zero correlation. In the present research, zebrafish preferred a dependent chasing motion over independent motion. Second, the present research indicated that preference could vary depending on the type of dependent motion. Specifically, both chasing and its corresponding control condition, the flipped animation, were dependent motions, but zebrafish preferred the chasing motion over the flipped motion. Therefore, the quality of the movement affects preference, with conspecific movement appearing to be preferred more when directed toward the experimental fish (i.e., chasing) than away (i.e., fleeing, flipped).

Another important implication of the present research was that operant conditioning might underlie preference for social stimuli. In typical operant-conditioning procedures, experimental subjects engage in discrete responses resulting in access to some outcome. For example, rats press a lever that results in response-dependent food delivery. Similar to the present findings with social stimuli, several species showed more responses in the presence of a response-food dependency than in its absence (e.g.^35–37^) including zebrafish^38^. One line of inquiry that follows from determining whether operant conditioning underlies preference for social stimuli is whether operant conditioning is implicated in the development of other social behavior, such as schooling.

An alternative account for many findings of preference, including the present data, is the increased proximity to one stimulus over another could reflect nonassociative processes, rather than operant conditioning. Specifically, preference could instead be an instance of an unconditioned approach response to stimulus features of conspecifics. The procedures used in the present experiments and many others to measure preference differ from typical operant-conditioning procedures with nonhumans that serve to identify reinforcing effects (e.g.^39–41^). In typical operant research, the availability of positive reinforcers (e.g., food delivery, drug, social interaction) is dependent upon and limited to immediately after the occurrence of an arbitrary target response (e.g., lever press). Therefore, operant responses are generally different from the behavior required to consume the reinforcer, and consumption of the reinforcer spans a protracted time period. In contrast, the behavior comprising the operant response to evaluate preference in zebrafish is not differentiated from behavior “consuming” the continuously available social reinforcer. A more direct approach to evaluating whether operant behavior comprises preference for movement in zebrafish would be to arrange a choice procedure between two discrete responses (e.g., photocells)^10^, in which responding on one alternative results in a dependent motion of a stimulus fish and the other alternative leads to an independent motion or other control condition, with each being available only to immediately after the respective response.

**Figure 6:**
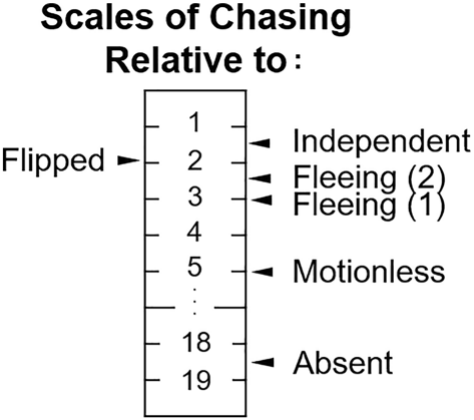
Preference scales of chasing relative to different types of motion. Values presented here are antilog of arithmetic mean log time ratios in Phase 2 of each condition presented in Figs. 4 and 5.

One limitation of the present research relates to the interpretation of time ratio as an index of preference. Figure 6 shows preference scales of chasing relative to different types of motion (cf.^42^). For instance, zebrafish prefer the chasing motion approximately five times more than motionless. This, however, should not be interpreted as that the fivefold difference necessarily will generalize to other situations. The magnitude of relative preference would most likely change, for example, when changing the length of aquarium from 45 cm to, say, 90 cm. Longer aquariums introduce greater costs of switching between left and right compartments^43,44^. In an extremely long aquarium, experimental fish might exclusively stay in one of the compartments. Thus, researchers should always consider variables within the experimental setup when interpretating quantitative measures of preference.

Another limitation of the present research is we examined the chasing contingency repeatedly throughout both experiments, thereby providing overall more exposure to chasing than other movements by stimulus fish. Repeated exposure to a single type of stimulus introduces the concern that preference could change with repeated exposure, through either habituation or sensitization^45^. However, we replicated the chasing versus fleeing condition during Experiment 2 and observed no reliable differences between exposures, suggesting no discernable influence of habituation or sensitization under these circumstances.

The present 3D animation method has potential for further examining processes underlying social behavior in zebrafish. A single female stimulus fish was presented on a monitor in the present research, but it can easily be extended to male zebrafish, predator fish, or multiple stimulus fish. Manipulating shoal size, a previous study showed that female zebrafish preferred larger shoals to smaller ones regardless of the composition of males and females, whereas males showed no preference^19^. Using the present method, multiple stimulus fish could be presented simultaneously while having each individual fish’s motion be dependent on an experimental fish’s motion to examine not only shoaling but also schooling. In addition, the degree of synchronization among schooling fish’s motions could also be precisely manipulated using 3D animations. These experimental manipulations were not possible in previous research using pre-recorded videos or robotic fish. Nevertheless, there were aspects of the 3D animations that could be improved upon to enhance realism. For example, our 3D animations did not include flexion of the body and tail typical of live and video-recorded fish. It is noteworthy then that zebrafish prefer a shoal of live conspecifics with interdependent motions compared with a video that would lack such interdependency^46^. Although the current methods improved upon the visual display from previous research^25^, further technological improvements to display more biologically realistic motion (e.g., smooth body, interactive movements) would further advance our understanding of factors contributing to social preference. Lastly, the present procedure could also be useful when examining effects of biological, developmental, and other environmental factors on social behavior. Genetic modifications (e.g.^17^), embryonic exposures to drugs (e.g.,^18^), and being reared alone during early developmental stages^47^, each result in abnormal social behavior in zebrafish. Moreover, even a short period (e.g., 48 h) of isolated housing can affect social preference^48^. Nonetheless, it remains unclear which aspect of social behavior is affected by these biological/developmental/environmental factors because the relation between motions of experimental and stimulus fish have been left uncontrolled in most studies, using either live conspecifics or pre-recorded videos. The present and previous research (e.g.^21,22^) together suggest that zebrafish social behavior can be affected not only by stimuli preceding their behavior but also by those consequences following it. Interdisciplinary research between biomedical and behavioral sciences could lead to better understanding of how biological and developmental factors affect social behavior.

## Data Availability

The datasets generated during and/or analyzed during the current study are available at https://github.com/ToshiEAB/social_stimulus_preference (dataset_social_stimulus_preference.xlsx).

## Code availability

Programs for experimental tasks and for data analyses included in this manuscript are available at https://github.com/ToshiEAB/social_stimulus_preference (Social_3D_Animation_PreferenceTest) and https://github.com/cmr0112/Zebrafish_Social_Preference (Rmd files).
